# The impact of hip fracture on mortality in Estonia: a retrospective population-based cohort study

**DOI:** 10.1186/s12891-017-1606-1

**Published:** 2017-06-05

**Authors:** Mikk Jürisson, Mait Raag, Riina Kallikorm, Margus Lember, Anneli Uusküla

**Affiliations:** 1 Institute of Family Medicine and Public Health, University of Tartu, Ravila st 19, 50411 Tartu, Estonia; 2 Institute of Clinical Medicine, University of Tartu, L. Puusepa st 8, 51014 Tartu, Estonia; 3 Internal Medicine Clinic, Tartu University Hospital, L. Puusepa st 8, 51014 Tartu, Estonia

**Keywords:** Hip fracture, Excess mortality, Eastern Europe, Estonia

## Abstract

**Background:**

Estimates of hip fracture mortality in Eastern Europe are scarce. We aimed to estimate the magnitude and duration of excess mortality after hip fracture in Estonia.

**Methods:**

Retrospective, population-based 10-year study of persons aged ≥50 in two cohorts: with hip fracture and an age- and sex-matched (in a 1:4 ratio) random sample from the national health insurance fund database for comparison. Cumulative risks, excess risks and relative risks of death were estimated using Poisson regression with 95% bootstrap confidence intervals (CI). Risks were adjusted for age and Charlson comorbidity index.

**Results:**

We identified 8298 (2383 men, 5915 women) incident hip fracture patients from 2005 to 2013 and 33,191 (9531 men, 23,660 women) individuals for the reference group. 5552 (1564 men, 3988 women) cases and 14,037 (3514 men, 10,523 women) reference individuals died during the 10-year follow-up period. Among hip fracture patients we observed a pronounced and durable excess risk of death that was highest within 3–6 months after fracture and persisted for the full 10-year follow-up period. After adjustment for age and Charlson index, hip fracture was associated with a 21.1% (95% CI 20.0–22.5%) 10-year cumulative excess risk of death (RR 1.37, 95% CI 1.35–1.40). We found a high immediate excess risk of death in older age groups (≥80 years) and gradually accumulating excess risk in younger age groups (50–79 years). The excess risk was more pronounced among men than women.

**Conclusions:**

By the end of the 10-year follow-up, 1 in 4 deaths in the hip fracture group was attributable to the hip fracture. The results indicate a high attributable impact of hip fracture as an independent risk factor for death.

## Background

Patients who have a hip fracture are at considerable risk for premature death [1, 2]. A recent report of osteoporosis in the European Union estimated that mortality related to low-impact trauma hip fracture is greater than road traffic accidents and equivalent to breast cancer [3]. This mortality burden will increase over the next few decades commensurate with the aging of the population [3]. Targeted interventions among at-risk groups may contribute to mortality reductions [1], thus, a contemporary epidemiology of hip fracture mortality would be useful in developing risk profiles and estimates of potential lives saved. To this end, country-specific mortality data should be collected to refine estimates of the longitudinal burden of hip fracture and conduct economic evaluations of hip fracture prevention and treatment measures [4].

The data describing excess mortality after hip fracture are well established in developed Western European and North American countries [1, 5]. In a systematic analysis, the documented cumulative excess risk (i.e., exceeding mortality rates among non-hip fracture or community control populations) during the first year after hip fracture varied widely from 8.4% to 36% [1]. Studies of short- vs. long-term mortality almost always note increased mortality soon after the fracture, within the first 3–6 months [1, 2, 5–9]. Although the relative risk decreases in subsequent years, it does not return to that of age- and sex-matched reference groups even 10 years post-fracture [5]. The excess risk increases with advancing age, although this differential becomes less pronounced over the following years due to increased mortality, unrelated to hip fracture, in the reference populations [1, 5]. The excess risk is also higher among men than women [1, 5], notably among the oldest age categories (≥80 years) in the first months and years after fracture [5].

Estimates of hip fracture mortality in Eastern Europe are scarce [10, 11]. However, the data in this region suggests a sex-specific difference in the incidence of hip fractures between Eastern and Western Europe [12–14], and the age and sex-specific all-cause mortality rates in Eastern Europe differ from those in Western countries [15]. Longitudinal data is needed to quantify the change in excess mortality after hip fracture by temporal, clinical and geographic characteristics. This study estimates the impact of hip fracture on 10-year all-cause mortality among Estonian men and women ≥50 years of age.

## Methods

### Overview

This was a population-based retrospective cohort study to examine the excess all-cause mortality after hip fracture in Estonia. The data on all-cause mortality in men and women aged ≥50 years with incident hip fracture (cases) were compared with randomly selected age- and sex-matched subjects from the reference group with no known history of hip fracture prior to the index date (defined below). The excess mortality risk related to the hip fracture over a 10-year follow-up period was evaluated using Poisson regression and adjusting for demographic and clinical characteristics.

### Setting, data source, and participants

The population of Estonia is approximately 1,315,000 residents, of whom 479,000 are aged 50 years or more (197,000 men and 282,000 women) (2015 data) [16], and universal public health insurance covers >94% of the population [17]. Since its inception in the early 2000s, the Estonian Health Insurance Fund (EHIF) has maintained a complete record of inpatient and outpatient health care services. The EHIF electronic database contains information on characteristics of the person (sex, age at health care service utilization), health care utilization (date of service, primary and other diagnoses, treatment type (in- or outpatient), a specialty of the provider), and the date of death. For the current study we ascertained study subjects’ demographic characteristics, clinical characteristics, and outcome data. Although the EHIF database captures provision of healthcare services country-wide, loss to follow-up upon emigration from Estonia is possible, albeit rare, among those 50 years or older (estimated to be less than 0.5% per year) (2015 data) [16].

The sample frame included all publicly insured individuals, including those with no record of health care services provided from January 1, 2004 – December 31, 2013. Health care utilization data on all patients (aged ≥50 years) hospitalized with incident hip fractures during the period January 1, 2005-December 31, 2013 were identified (case group). The hip fracture patients were matched by sex and age (year of birth) in a 1:4 ratio to the reference group. By definition, reference group subjects were alive and without evidence of hip fracture prior to the case patient’s index date of fracture. Study subjects were assigned a unique identifier decoupled from personal identification information to enable longitudinal tracking of care and mortality while maintaining patient privacy. We followed all study subjects (belonging to the case and reference groups) until the study closure May 4, 2016, or the date of death.

#### Identification of incident hip fracture

The case definition of hip fracture was based on the following specific diagnosis codes (The International Classification of Diseases, Tenth Revision (ICD-10): S72.0 - fracture of femoral neck, S72.1 - pertrochanteric fracture and S72.2 - subtrochanteric fracture). These codes must have been listed as the primary diagnosis on the electronical inpatient health care claim submitted to the EHIF. The index date of diagnosis was defined as the first day of care indicated in the claim; patients with a diagnostic code primary for hip fracture and no previous evidence of hip fracture according to EHIF records were selected for inclusion. The validity of this case definition (i.e., incident hip fracture case) from a population-based administrative database has been demonstrated [18].

#### Identification of pre-fracture comorbidity

Clinical characteristics and comorbidities were captured for the 365-day period prior to the index date for case patients and their matched controls. The comorbidity status for both groups was computed using the Charlson comorbidity index (CCI) [19]. We used the revised coding algorithm described by Quan, et al., and subsequently validated for estimating comorbidity using ICD-10 coded administrative data [20]. We also updated disease weighting to reflect advances in chronic disease management and treatment outcomes since the introduction of the original Charlson index in 1984 [21]. The updated Charlson index has demonstrated comparable predictive utility for mortality using ICD-10 coded administrative data [21] and has been validated among hip fracture patients [22].

Comorbidities were defined as secondary or other diagnoses coded at the index hip fracture claim and/or diagnoses of any type on hospital or outpatient health care claims during the year preceding the index date [22, 23]. We applied a restriction to outpatient claims, such that a comorbid condition could be flagged during the preceding period only if it appeared two or more times at least 7 days apart [7, 23].

The primary outcome for the study was death from any cause. The dates of death were obtained from the EHIF database.

### Statistical analysis

The hip fracture group and the reference group were described by group size, mean age, age range and 10-year groups, CCI mean score and range; 95% Wald confidence intervals for mean differences of age and CCI were calculated. To capture the rapid and extensive changes in mortality during the first 3--6 months following a hip fracture [1, 5], we divided our 10-year follow-up period into graduated discrete intervals as follows: using shorter periods close to the index date and wider intervals later with cut-points at 0.5, 1, 1.5, …, 3, 4, …, 12, 15, …, 24, 30, …, 48, 60, …, 120 months after the index date. Subsets defined by age group and sex were analyzed separately, the results were aggregated by weighting by group sizes when necessary. Within each subset Poisson regression was used to estimate mortality rates for each of those intervals. Two regression models were considered: crude (unadjusted), containing only the interaction between hip fracture and follow-up time interval besides main effects of both and the logarithm of time at risk as an offset term, and adjusted, including main effects of hip fracture, CCI, age, follow-up time interval, and logarithm of time at risk in the respective follow-up time interval as an offset term as well as interactions between hip fracture and follow-up time interval, hip fracture and CCI, hip fracture and age, follow-up time interval and age. Interval-specific mortality rates were transformed to probabilities which were used to calculate cumulative risks. Age adjustment within age groups was used to account for residual confounding [24]. Excess risks and risk ratios (RR) were calculated using cumulative risks. Bootstrap with bias correction was used to compute 95% confidence intervals (CI) for cumulative and excess risks, and the bootstrap percentile method was used to calculate 95% CIs for risk ratios.

All analyses were performed in R (versions 3.1.1 to 3.3.1) [25–32].

The study procedures were conducted in accordance with local data protection regulations. The study was approved by the Tartu University Research Ethics Committee.

## Results

### Characteristics of hip fracture patients and reference group subjects

From the EHIF database we identified 8298 (2383 men, 5915 women) incident hip fracture patients and 33,191 (9531 men, 23,660 women) individuals for the reference group between 2005 and 2013 (Table 1). There were only three reference individuals available for one 100-year old man with hip fracture, therefore the total size of age- and sex-matched reference (non-hip fracture) group was 33,191. Similar numbers of hip fractures occurred each year in 2005—2013. Most of the fractures (71%) occurred in women. On average, men were 8.2 years (95% CI 7.7—8.7) younger than women at hip fracture, over 70% of men were younger than 80 years at hip fracture while 60% of women were 80 years or older.

**Table 1:** Characteristics of hip fracture patients and reference group subjects aged 50 years or more in Estonia, January 1, 2005, to December 31, 2013

Characteristic	Hip fracture group				Reference group			
	Men		Women		Men		Women	
	n	%	n	%	n	%	n	%
N	2383	29%	5915	71%	9531	29%	23,660	71%
Age, mean (sd)	72.2 (11.4)		80.4 (9.1)		72.2 (11.4)		80.4 (9.1)	
Age range	50–102		50–103		50–102		50--103	
Age groups								
50–59	401	17%	202	3%	1604	17%	808	3%
60–69	561	24%	484	8%	2244	24%	1936	8%
70–79	724	30%	1689	29%	2896	30%	6756	29%
80–89	564	24%	2749	46%	2256	24%	10,996	46%
90+	133	6%	791	13%	531	6%	3164	13%
Charlson index, mean (sd)	0.88 (1.38)		0.96 (1.36)		0.59 (1.11)		0.69 (1.13)	
Charlson index range	0--9		0--9		0--9		0--10	

Mean Charlson comorbidity index was 0.28 points lower (95% CI 0.24—0.31) among the reference group (mean 0.66, SD 1.12) than in the hip fracture group (mean 0.94, SD 1.36), this difference was similar both among men and women (Table 1).

### Absolute risk of death

**Fig. 1:**
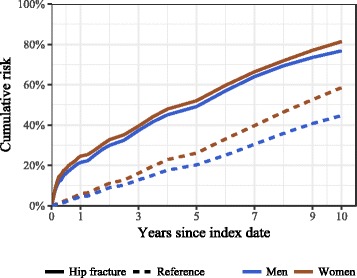
Sex-specific cumulative 10-year risk of all-cause mortality (adjusted for age and Charlson index score) by study group in men and women ≥50 years in Estonia, January 1, 2005-May 4, 2016

**Fig. 2:**
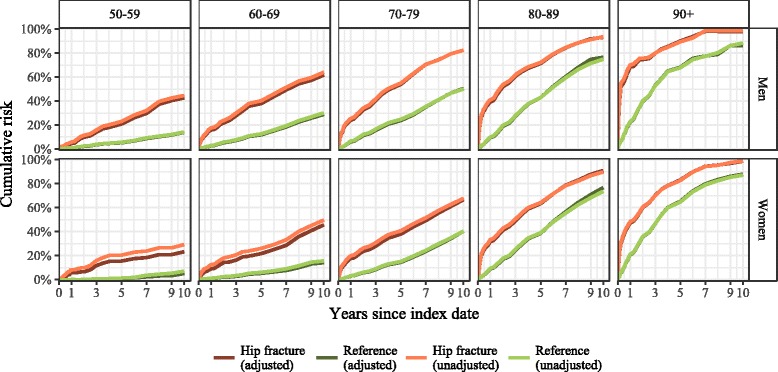
Age group-specific cumulative risk of all-cause mortality (adjusted for age and Charlson index score) by study group in men and women ≥50 years in Estonia, January 1, 2005-May 4, 2016

The average follow-up time was 4.3 years (3.4 years among hip fracture patients and 5.0 among the reference group. 5552 cases (1564 men, 3988 women) and 14,037 reference individuals (3514 men, 10,523 women) died during the 10-year follow-up period. The crude cumulative risk of death in the hip fracture group was high compared to reference patients: at 3 months 17.5% (95% CI 16.8–18.1%) vs 2.0% (95% CI 1.9–2.1%), respectively, in 1 year 28.3% (95% CI 27.6–29.0%) vs 7.8% (95% CI 7.6–8.0%), in 5 years 54.4% (53.6–55.2%) vs 29.8% (95% CI 29.4–30.1%), and in 10 years 78.2% (95% CI 77.2–79.2%) vs 55.6% (95% CI 55.0–56.2%), respectively. When stratified by sex, women experienced an elevated crude cumulative 10-year risk of all-cause death among both the hip fracture and reference groups (both *p* < 0.0001). The adjusted cumulative 10-year risk of all-cause death was 77.6% (95% CI 76.7–78.8%) in the hip fracture group and 56.5% (95% CI 56.0–57.3%) in the reference group. The adjusted sex-specific cumulative risks in both study groups are presented in Fig. 1, and the adjusted age-specific cumulative risks are presented in Fig. 2.

### Excess (attributable) risk of death

We observed a pronounced and durable excess risk of death after a hip fracture that was highest within 3–6 months after fracture and persisted for the full 10-year follow-up period. Compared with reference group individuals, those with hip fracture experienced 22.6% (95% CI 21.4–23.8%) crude excess risk, and 21.1% (95% CI 20.0–22.5%) adjusted excess risk in 10-year cumulative mortality. The proportion of deaths in the hip fracture group attributable to the exposure (attributable risk fraction) in 10 years from fracture was 27.2% (95% CI 25.9–28.5%). Consequently, by the end of the 10-year follow-up, one in four deaths in the case group was attributable to the patient’s hip fracture. 10-year attributable fraction varied widely across the age and sex groups: in the 50–59-year age group it was 67.5% (95% CI 61.1—73.1%) in men and 78.0% (95% CI 61.5—87.6%) in women, in the 70–79-year group 38.8% (95% CI 34.7—42.3%) and 39.2% (95% CI 35.8—42.6%), whereas in over 90-year old group it was only 13.0% (95% CI 7.4—20.3%) in men and 11.1% (95% CI 8.9—13.2%) in women.

**Fig. 3:**
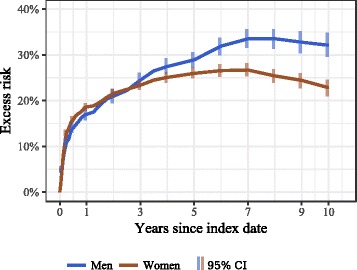
Excess cumulative 10-year risk of all-cause mortality following hip fracture among men and women age ≥ 50 years (adjusted for age and Charlson index score) in Estonia, January 1, 2005-May 4, 2016

The age- and CCI-adjusted excess cumulative risk of death for hip fracture group patients is presented in Fig. 3. In stratified analysis, the adjusted excess risk was extensive within 3 months of the fracture (13.7%, 95% CI 12.9–14.9% in men, 14.5%, 95% CI 13.8–15.3% in women), and increased by 1 year to 18.7% (95% CI 17.8–20.2%) in men and 19.2% (95% CI 18.3–20.2%) in women. The excess risk was highest by the seventh year after hip fracture in men (28.5%, 95% CI 26.8–30.3%) and by the fifth in women (21.9%, 95% CI 19.5–21.9%). After 3 years of the hip fracture, men were at significantly greater excess risk of death than women.

**Fig. 4:**
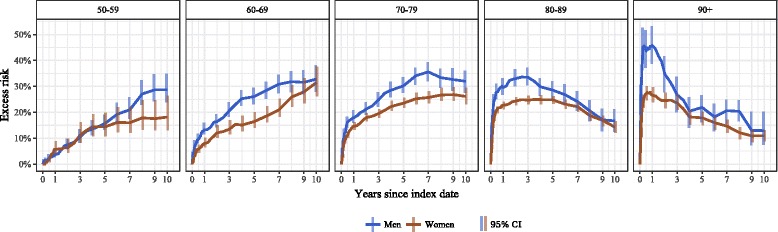
Age group-specific 10-year cumulative excess risk of death following hip fracture among those ≥50 years old (adjusted for age and Charlson index score), men and women in Estonia, January 1, 2005-May 4, 2016

The adjusted excess risk of mortality in the hip fracture group increased exponentially with age immediately after fracture (Fig. 4). For example, in the 60–69-year-olds, the 3-month excess risk was moderate (men 8.1%, 95% CI 6.5–9.9%; women 4.6% (95% CI 3.3–6.2%), whereas in the ≥90-year group the risk was 45.1% (95% CI 38.7–52.7%) in men and 24.2% (95% CI 21.8–26.7%) in women. However, over the study period the excess risk accumulated differentially by age. In older patients (≥80 years) the excess risk decreased over the observation period, but in younger age groups (50–79 years) the excess risk increased in a linear fashion over the 10-year follow-up period. For example, in the 60–69-year group the excess risk increased to 30% in 10 years (men 32.8%, 95% CI 28.0–38.1%, and women 31.5%, 95% CI 26.1–37.6%). In all age- and sex-specific groups, the excess risk of mortality due to hip fracture was present until the end of the follow-up period, and in any given age group the excess risk was higher in men.

### Relative risk of death

The adjusted relative risk of all-cause death among hip fracture patients versus age- and sex-matched controls is presented in Table 2. At 1 year, the hip fracture patients were between two and 10 times more likely to die than their age- and gender-matched reference subjects, depending on age and sex. The long-term relative risk was higher in younger age groups (women greater than men) where the absolute risk in the respective reference groups was lower and decreased with advancing age. The relative risk remained elevated over 10 years in all age- and sex-specific comparisons.

**Table 2:** Age group specific and average 10-year relative risk (risk ratio (RR) comparing hip fracture cases to reference group) of all-cause death after hip fracture in men and women ≥50 years, adjusted for age and Charlson index score

Sex	Age group	3 months		1 year		5 years		10 years	
		RR	95% CI	RR	95% CI	RR	95% CI	RR	95% CI
Men	50–59	*		5.6	3.3–10.2	4.0	3.2–4.9	3.1	2.6–3.7
	60–69	10.6	7.3–18.1	5.9	4.8–7.5	3.2	2.9–3.6	2.1	1.9–2.4
	70–79	10.0	7.8–13.2	3.9	3.4–4.5	2.3	2.1–2.4	1.6	1.5–1.7
	80–89	9.9	8.2–12.4	4.0	3.6–4.6	1.7	1.6–1.8	1.2	1.2–1.3
	90+	8.0	6.3–11.0	3.1	2.7–3.7	1.3	1.3–1.4	1.2	1.1–1.3
	Weighted average	9.5	8.4–10.9	4.1	3.8–4.4	2.0	2.0–2.1	1.6	1.5–1.6
Women	50–59	*		*		14.6	7.7–44.0	4.6	2.6–8.1
	60–69	14.6	8.6–34.8	9.8	6.8–15.0	4.1	3.4–5.0	3.3	2.8–3.9
	70–79	14.9	11.5–19.3	6.0	5.3–6.8	2.6	2.5–2.8	1.6	1.6–1.7
	80–89	9.1	8.2–10.1	3.5	3.3–3.7	1.7	1.6–1.7	1.2	1.2–1.2
	90+	5.3	4.6–6.1	2.3	2.1–2.5	1.3	1.2–1.3	1.1	1.1–1.2
	Weighted average	8.3	7.7–9.0	3.4	3.3–3.6	1.7	1.7–1.8	1.3	1.3–1.3

*Respective risk ratios had too high variance and were not reliable

## Discussion

Our study assessed the impact of hip fracture on the all-cause risk of death over 10 years among people 50 years of age or older in Estonia. We observed a pronounced and sustained excess mortality risk after a hip fracture that was highest within 6–12 months after the fracture and persisted for the full 10-year follow-up period. After adjustment for age and pre-fracture comorbidities (CCI), hip fracture was associated with a 21.1% (95% CI 20.0–22.5%) 10-year cumulative excess risk of death (RR 1.37, 95% CI 1.35–1.40). Even after 10 years following the hip fracture, more than 1 in 4 deaths among hip fracture patients was attributable to the initial injury. The 1-year average relative all-cause excess mortality (4.1 in men, 3.4 in women) after hip fracture was comparable to that of dementia, cancer, and heart failure [21], mental disorders [33], and higher than that for diabetes [34].

The excess risk of death differed by the duration of follow-up time after the hip fracture, by sex, and according to age at the time of fracture. We found a high immediate excess risk of death in older age groups (≥80 years) and a gradually increasing excess risk in younger age groups (50–79 years) that was more pronounced in men than in women. In the elderly, hip fracture has an immediate devastating impact on mortality that lasts for years. For example, in the group of men ≥90 years old, the excess risk of death 3 months after fracture was 45%, and the risk of dying was 8 times higher than in men who had not had a fracture. Consequently, over half of the men in that group had died within 3 months of the fracture, and within 12 months over two-thirds of the men had died. It is important to note that the excess risk among the elderly (aged 80 years or more) persisted throughout the 10-year follow-up period and did not disappear in any age- or sex-specific group. In contrast, the excess risk in younger age groups increased in a linear fashion over the follow-up period. For example, in the 60–69-year group the excess risk increased with time to a maximum of 30%. Thus, 6 out of 10 men with a hip fracture and 1 in 2 women in that age group died during the 10-year follow-up period. The risk of death was 2–3 times higher in the fracture group than in the reference group even 10 years after the fracture.

Previous studies have demonstrated an immediate elevated risk of mortality after hip fracture [1, 2, 5, 6, 35–39], however, the evidence of persistence is not universal [1, 7, 9, 40, 41]. Overall, our results are in line with the meta-analysis suggesting that the excess mortality after hip fracture in patients over 50 years is extensive already in the first months after fracture and persists for at least 10 years [5]. Notably, the relative risk of all-cause mortality within the 3 months after hip fracture was as high as 9.5 in men and 8.3 in women, comparable to that in the meta-analysis [5]. However, compared to the pooled estimates [5] the excess risk of death in Estonian men and women in younger (50–79 years) age groups was rather high, particularly in the first months and years after fracture. For example, in the 70–79-year-old men the excess risk in our study reached as high as 18% within 1 year, and 30% within 5 years, whereas in the meta-analysis the respective estimates were lower (11% and 20%). Likewise, in women of the same age we found the excess risk to be 14% in 1 year and 24% in 5 years, versus 5% and 13% in the meta-analysis. It is difficult to explain the reasons for increased mortality in these groups, but insufficient case management upon discharge and low utilization of rehabilitation, nursing care, and social care [42] could be potential contributors. In addition, it is possible that not all comorbid conditions were diagnosed and recorded in these patients (especially men), and their impact on mortality therefore could not be adjusted in the models. Finally, excess mortality study results are difficult to compare due to differences in study design and sources of data, ascertainment of cases and controls, determination of death, differences in follow-up time, presentation of results, and adjustment for confounding [1, 5].

A number of confounding factors, such as advanced age, male sex, poor preoperative health status and multiple comorbidities have been associated with excess mortality following hip fractures [43]. Our results are in line with the collective evidence confirming that excess mortality increases with age, and is higher in men than in women in all age groups [1, 5]. The average excess risk among men in our study did not exceed that among women during 3 years following the fracture; this may be explained by the different age distribution of fractures in men and women in our study. We know that most hip fractures in Estonian men occur at a younger age (50–79 years) due to the elevated incidence of hip fractures in that age [14] and low life expectancy in men (15.1 years at age 65, 2014 data) [16], whereas over half of fractures in women occur among those ≥80 years [14]. Due to the considerable age difference between sexes in our study (8.2 years) women experienced an elevated crude cumulative 10-year risk of all-cause death among both the hip fracture and reference groups (see Fig. 1), and the weighted average excess risk in both study groups (see Fig. 3) was influenced by the higher-weighted age groups, with younger groups in men and older groups in women.

Numerous studies have reported that the presence of pre-fracture concomitant medical conditions are negative predictors for survival [35, 36, 43–46], whereas the extent to which underlying conditions contribute to the excess mortality associated with hip fracture is still unclear [1]. In our study the hip fracture patients had higher CCI score than the age- and sex-matched reference subjects. However, because the sample in our study was matched for age and sex, and the risks were adjusted for major confounders, we believe that the results strongly suggest that hip fracture is an independent and attributable risk factor for death. This implies that preventative efforts and post fracture rehabilitation and social care are essential to reduce the excess risk of death.

The possible reasons for the greater mortality risk in men than in women following hip fracture are still poorly understood [1]. Previously described risk factors in older men include multi-morbidity, smoking, lower dietary protein, greater height combined with the use of antidepressants leading to a greater impact upon falling, whereas the traditional risk factors in women (rheumatoid arthritis, use of benzodiazepines and corticosteroids) were not related to hip fractures in men [47]. It has also been suggested that men have higher rates of pneumonia and septicemia than women [48], or more severe medical comorbidities prior to the hip fracture [49, 50]. However, in our study the CCI score was lower in men than in women in both study groups, suggesting that men were healthier than women. It is possible that the lower CCI score in men was related to their younger age compared to women. Our study adjusted for CCI, yet the excess risk was higher in men than in women.

We acknowledge the limitations of our study. Our objective was strictly aligned with assessing the impact of hip fracture on mortality, thus, we did not assess the impact of hip fracture complications on excess mortality, or the causes of death. Nursing home or facility residence, poor preoperative walking capacity, poor activities of daily living and poor mental state have been identified as strongly predictive factors for the excess mortality [43] suggesting that frail and disabled elderly are at higher immediate risk of death after hip fracture [7, 46]. It is possible that the level of functional impairment in our study was higher among hip fracture patients. We used data from the (administrative) health insurance database that covers the overwhelming majority of the Estonia’s population. However, we are not aware of any data documenting the completeness of the database. Furthermore, the data on additional useful indicators (sociodemographic factors such as income, education, occupation, social deprivation, and other health/lifestyle indicators (BMI, smoking, alcohol consumption)) [6, 43, 46, 51] were not available. Finally, we did not account for changes in hip fracture mortality in the population over time.

We also did not analyze in detail the impact of comorbidities on excess mortality in detail. We used the CCI as a well-accepted comorbidity burden index for adjusting for concomitant diseases [22, 23, 44, 45, 52, 53]. We chose the CCI because of its adaptability to large population databases using diagnostic codes from the ICD-10 [44]. It has also been documented that excess deaths among hip fracture patients can mainly be explained by the conditions predominantly responsible for mortality in the general population, i.e. those represented in the CCI [54]. However, we know that as a composite index it does not discriminate well between diseases, i.e. it equates the entities. Models incorporating comorbidities as individual variables perform better in predicting mortality than the weighted index [22]. The CCI ignores most of the disorders known to cause secondary osteoporosis [54], and it does not include hyperthyroidism or Parkinson’s disease which are known to increase the propensity to falls [47]. Furthermore, it does not allow for risk stratification based on disease severity [22, 44]. Further research is needed to identify the specific diseases most responsible for the excess mortality.

It is possible that the excess risk of death in our analysis was slightly overestimated due to measurement bias. Our data collection started from 2004, and some subjects with unascertained fractures before 2004 might have been misclassified as incident cases (fracture group) or non-fracture patients (reference group). This misclassification might have resulted in slightly overestimating mortality in both groups. However, as the risk for further hip fracture after previous hip fracture is over 2-fold [3, 55] and a subsequent fracture is associated with increased mortality risk [56], the overestimation would have been higher in the fracture group, resulting in a slightly overestimated excess mortality.

The comorbidity data for both groups were collected at the time of, and for 1 year before, the index dates of a hip fracture cases. We are aware that by using hospitalization data as a part of index case definition we might have introduced differential misclassification into assessing co-morbidity. However, we believe that people with severe life-threatening conditions would have received health care, and in including data from hospitalization episodes (including primary and secondary diagnoses) within the 12-months recall period into CCI for individuals in the reference group might mitigate some of this bias. Further, we speculate that potential differential misclassification described above might lead to overestimating the effect of comorbid conditions on mortality and thus support our main finding of hip fracture as a major independent risk factor for death.

To our knowledge, this is the first population-based observational study to estimate the impact of hip fracture on mortality in Eastern Europe. The strength of our analysis lies in the use of a data source with nationwide coverage (EHIF data). We had a large sample size of a representative population (given the >94% population coverage of the EHIF), long follow-up, and standardized recording of health events across the period of observation, which avoids problems related to imperfect recall and incomplete records. The large sample size provided a high number of events (deaths) to derive precise estimates over the long follow-up period, and the high frequency of observations allowed for assessment of rapid and extensive changes during the first months after fracture. We believe that our study provides informative results allowing inferences to other Eastern European hip fracture populations ≥50 years of age.

## Conclusion

This population-based study is a contemporary evaluation of the impact of hip fractures on mortality in Eastern Europe and adds to the rather scant data previously available. By the end of the 10-year follow-up, 1 in 4 deaths in the hip fracture group was attributable to the hip fracture. We found a high immediate excess risk of death in older age groups (≥80 years) and gradually accumulating excess risk in younger age groups (50–79 years), that was more pronounced in men than in women. Compared to the pooled estimates, the excess risk of death in younger (50–79 years) Estonian men and women was higher, particularly in the first months and years after fracture. The results indicate an attributable impact of hip fracture as a major independent risk factor for death, and suggest that preventive efforts and post fracture rehabilitation and social care are essential to reduce the excess risk of death. To reduce the excess mortality following hip fracture, research should focus on refining country- or region-specific prognostic indicators.

## Abbreviations

CCI: Charlson Comorbidity Index
EHIF: Estonian Health Insurance Fund
ICD-10: The International Classification of Diseases, Tenth Revision
SD: Standard deviation
