# Erratum to: The impact of hip fracture on mortality in Estonia: a retrospective population-based cohort study

**DOI:** 10.1186/s12891-017-1735-6

**Published:** 2017-08-24

**Authors:** Mikk Jürisson, Mait Raag, Riina Kallikorm, Margus Lember, Anneli Uusküla

**Affiliations:** 1 Institute of Family Medicine and Public Health, University of Tartu, Ravila st 19, 50411 Tartu, Estonia; 2 Institute of Clinical Medicine, University of Tartu, L. Puusepa St 8, 51014 Tartu, Estonia; 3 Internal Medicine Clinic, Tartu University Hospital, L. Puusepa St 8, 51014 Tartu, Estonia

## Erratum

After the publication of the article [1] it came to our attention that the wrong versions of Figs. 1 and 3 were published. Please find below the correct version of Figs. 1 and 3.

**Fig. 1:**
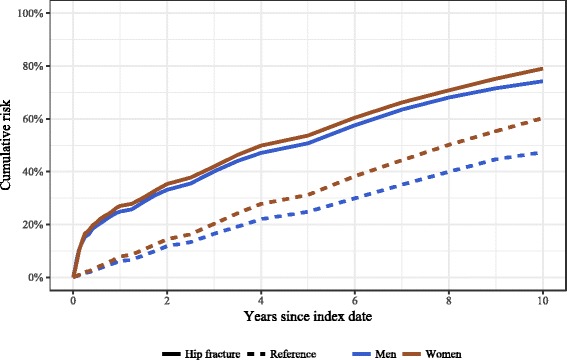
Sex-specific cumulative 10-year risk of all-cause mortality (adjusted for age and Charlson index score) by study group in men and women ≥50 years in Estonia, January 1, 2005-May 4, 2016

**Fig. 3:**
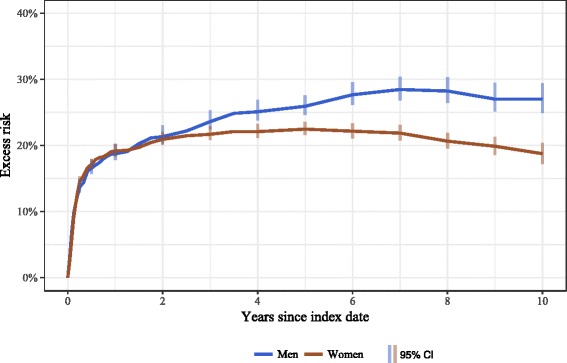
Excess cumulative 10-year risk of all-cause mortality following hip fracture among men and women age ≥ 50 years (adjusted for age and Charlson index score) in Estonia, January 1, 2005-May 4, 2016
